# Unusual synchronous double primary treatment-naïve lung adenocarcinoma harboring T790M and L858R mutations in early-stage lung cancer

**DOI:** 10.1186/s12957-019-1688-3

**Published:** 2019-08-19

**Authors:** Ching-Fu Weng, Po-Ju Chen, Ailun Heather Tseng, Shih-Hung Huang, Henry Hsin-Chung Lee

**Affiliations:** 1 Department of Thoracic Medicine, Hsinchu Cathay General Hospital, Hsinchu, Taiwan; 2 Department of Thoracic Surgery, Xizhi Cathay General Hospital, New Taipei, Taiwan; 3 Systems Biology and Bioinformatics, National Central University, Taoyuan, Taiwan; 4 Department of Pathology, Cathay General Hospital, No. 280, Sec. 4, Ren’ai Rd., Da’an Dist., 106 Taipei, Taiwan; 5 Department of Surgery, Hsinchu Cathay General Hospital, No. 678, Sec. 2, Zhonghua Rd., East Dist., Hsinchu City, 300 Taiwan

**Keywords:** Synchronous multiple primary non-small cell lung cancer, SMPNSCLC, Epidermal growth factor receptor

## Abstract

**Background:**

Concurrent mutations of synchronous multiple primary non-small cell lung cancer (SMPNSCLC) is rare, and only a few cases have been reported. Herein, we present a case of early-stage SMPNSCLC with T790M and L858R mutations.

**Case presentation:**

A 68-year-old male patient presented to the Thoracic Surgery Department due to a tumor in the right lower lung. The tumor was detected more than 5 years previously during a health examination; however, the patient ignored the problem because the clinician at that time stated that the lesion was highly likely to be benign. Chest computed topography (CT) was ordered and the images showed a well-defined tumor in the right lower lung and a faint nodular lesion over the left lower lung field. A CT-guided biopsy results showed the presence of atypical cells and positive staining of TTF-1 and CK7. Surgical intervention was performed. The right- and left-sided tumors disclosed micropapillary predominant adenocarcinoma and acinar-predominant adenocarcinoma, respectively. Both tumors were positive for TTF-1 but negative for ALK and p40. Real-time PCR analysis showed that the right-sided tumor had an epidermal growth factor receptor (EGFR) mutation presenting as point mutation T790M in exon 20, while the left-sided tumor had a point mutation L858R in exon 21 of EGFR.

**Conclusions:**

Our patient’s case suggests that tumors resembling a benign pattern with central calcification may be misdiagnosed. Thus, early screening for lung cancer is important, and intensive efforts to make a diagnosis through surgical resection or biopsies to allow for tailored optimal treatment may be preferential for the best patient outcomes.

## Introduction

Multiple primary lung cancer (MPLC) is defined as two or more primary lung cancers occurring in the same patient and can be categorized into synchronous multiple primary lung cancer (SMPLC) or metachronous multiple primary lung cancer (MMPLC) based on the time of occurrence [1]. The incidence of MPLC ranges from 0.2 to 20% [2, 3]. Li et al. reported that around 1.85% of patients with non-small cell carcinoma (NSCLC) had stage I synchronous multiple primary non-small cell lung cancer (SMPNSCLC) [1]. The most commonly reported histologic finding of SMPNSCLC is adenocarcinoma [4], which is consistent with the clinical phenomenon of a high proportion of adenocarcinoma in NSCLC. Previous studies have reported that the two main pathological types are adenocarcinoma in situ (AIS) and minimally invasive adenocarcinoma (MIA) in SMPNSCLC [5]. The distinct pathogenesis of lung cancer has yet to be elucidated, although somatic or germline mutations are considered to be genetic factors that drive the development of lung adenocarcinoma [6, 7]. Herein, we report an unusual case diagnosed with early-stage SMPNSCLC with treatment-naïve T790M- and L858R-mutant NSCLC in separate lung tumors, indicating the variety of genetic expression in the early stages of lung cancer, and highlighting the importance of a prompt pathologic diagnosis and surgical intervention to improve patient outcomes.

## Case report

**Fig. 1:**
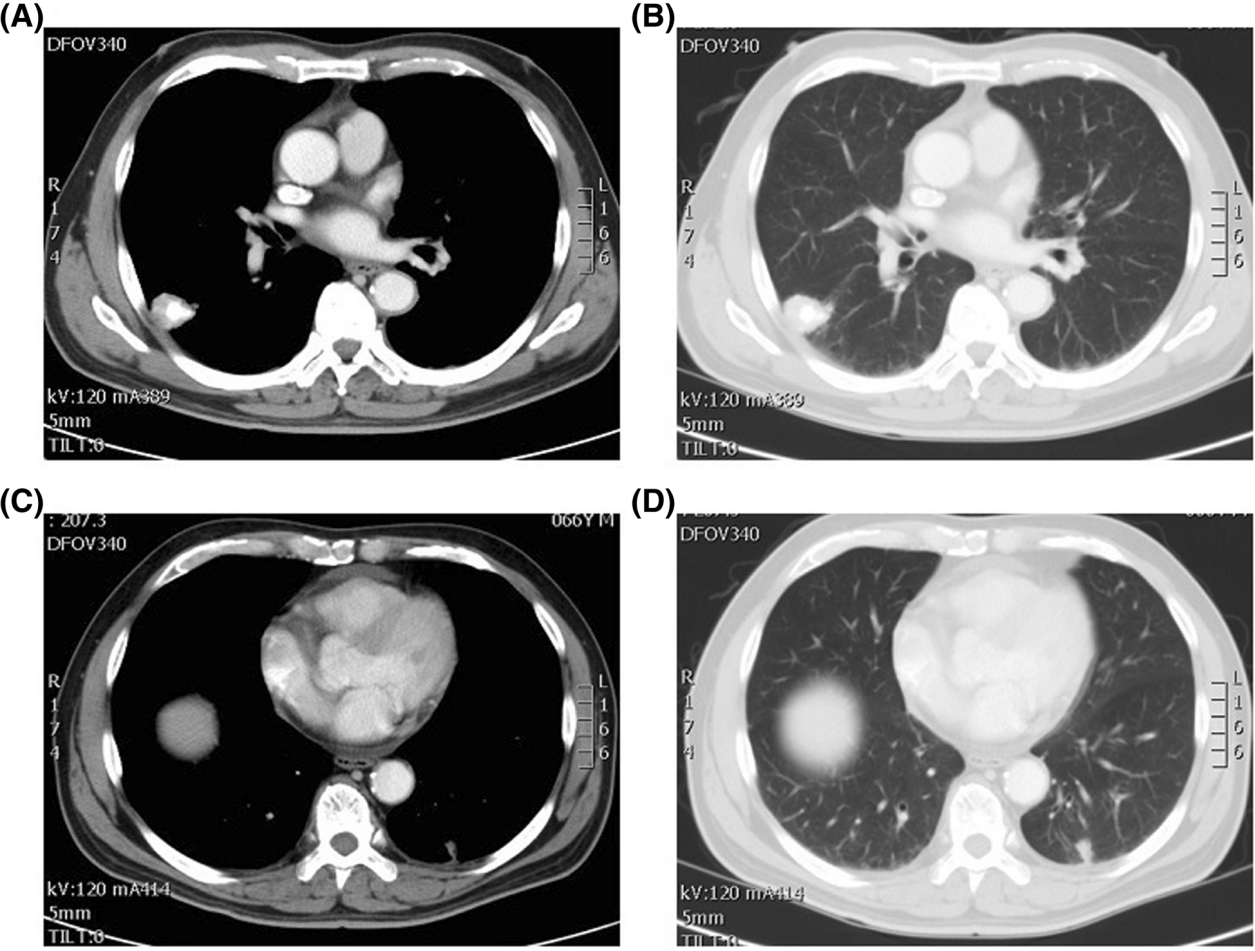
Synchronous multiple primary lung cancer of the 68-year-old patient. Chest computed tomography showing **a** right lower lobe mass (axial view), **b** right lower lobe mass (lung window view), **c** left lower lobe mass (axial view), **d** left lower lobe mass (lung window view)

**Fig. 2:**
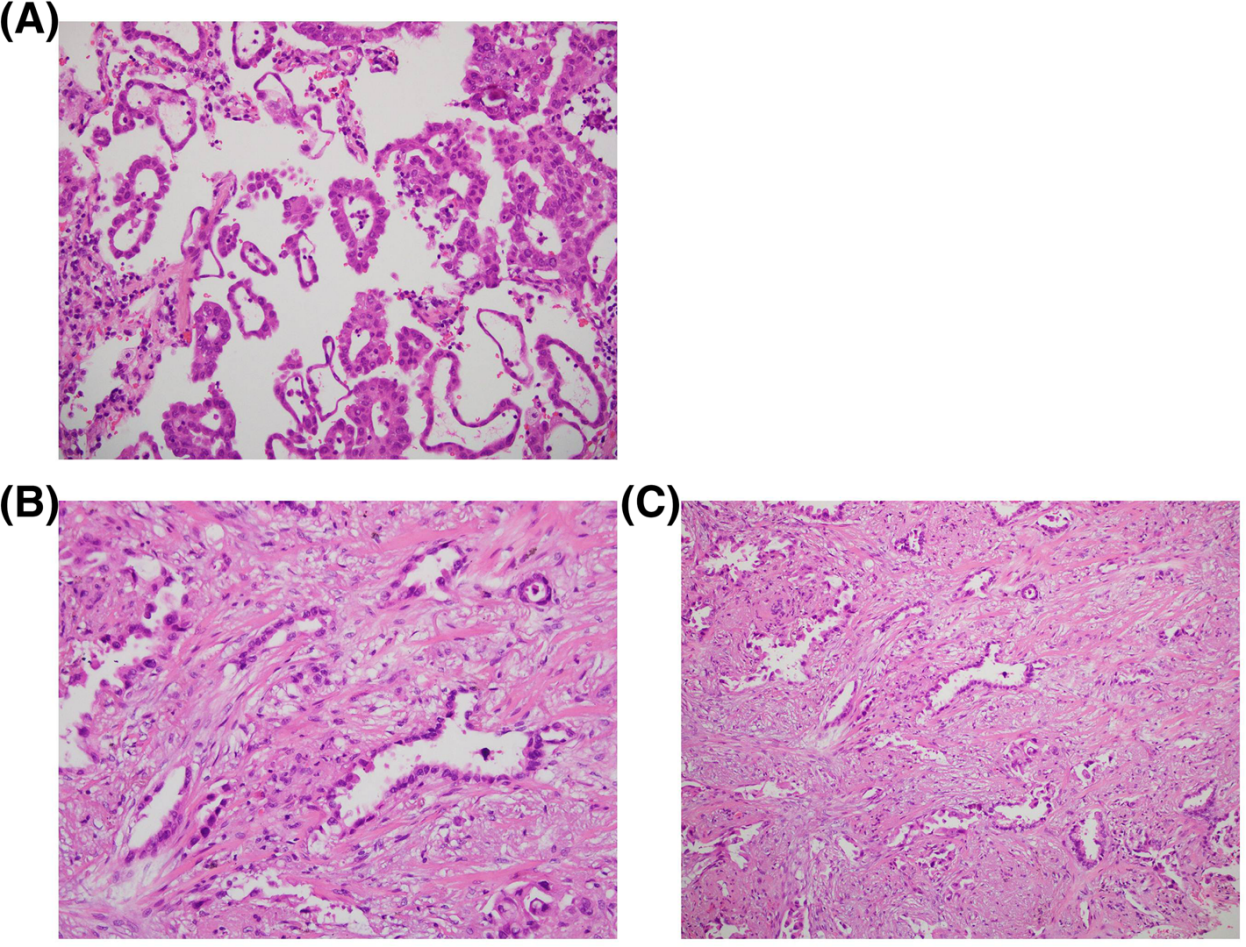
Histological features of the synchronous multiple primary lung cancer. **a** The right lower lung showed micropapillary predominant adenocarcinoma (H&E, × 200). **b** The left lower lung showed acinar-predominant adenocarcinoma (H&E, × 200). **c** The left lower lung showing acinar-predominant adenocarcinoma (H&E, × 100)

**Fig. 3:**
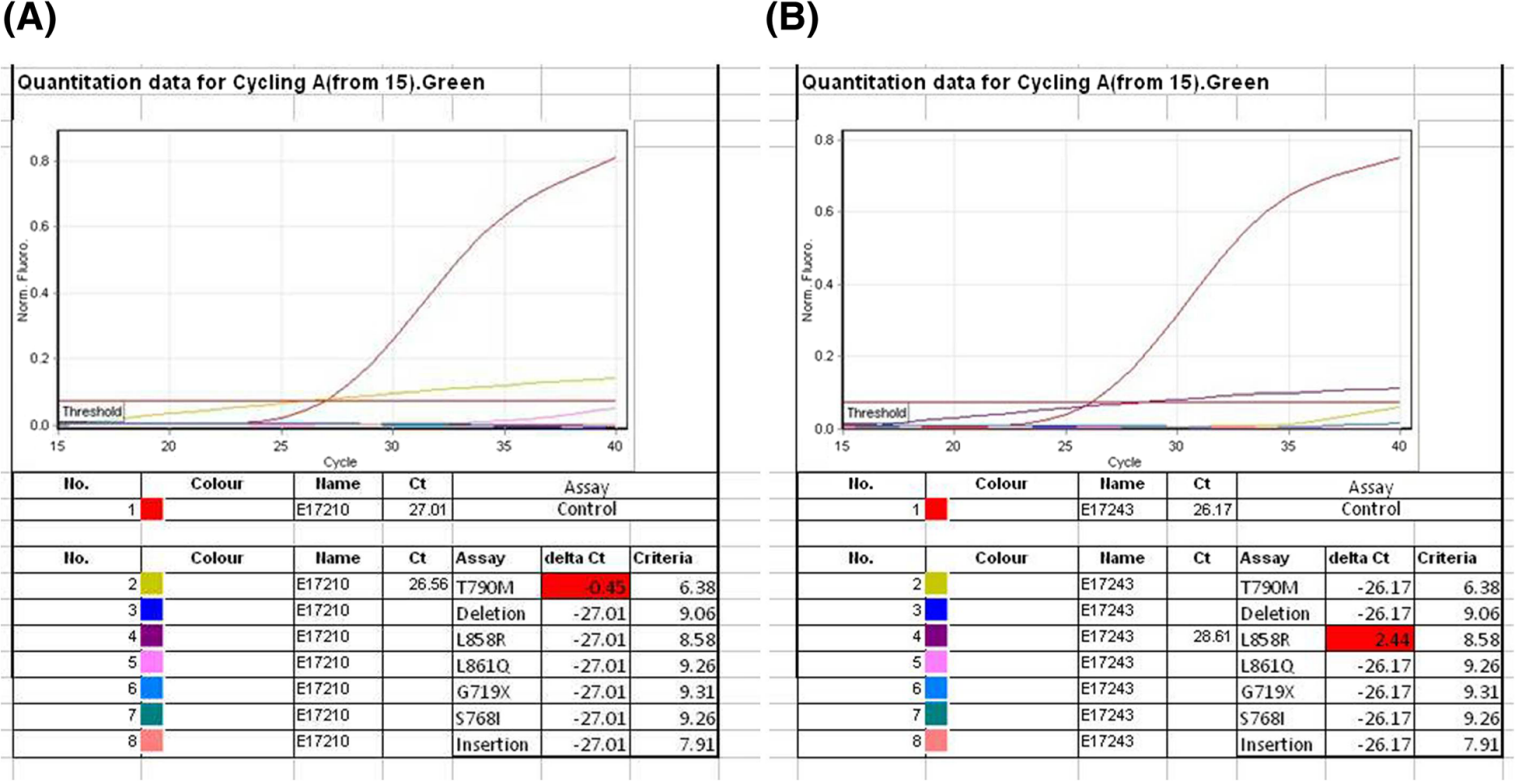
Real-time PCR analysis of EGFR mutation of adenocarcinoma. **a** EGFR point mutation T790M in exon 20 in the right lower adenocarcinoma. **b** EGFR point mutation L858R in exon 21 in the left lower lung adenocarcinoma

**Fig. 4:**
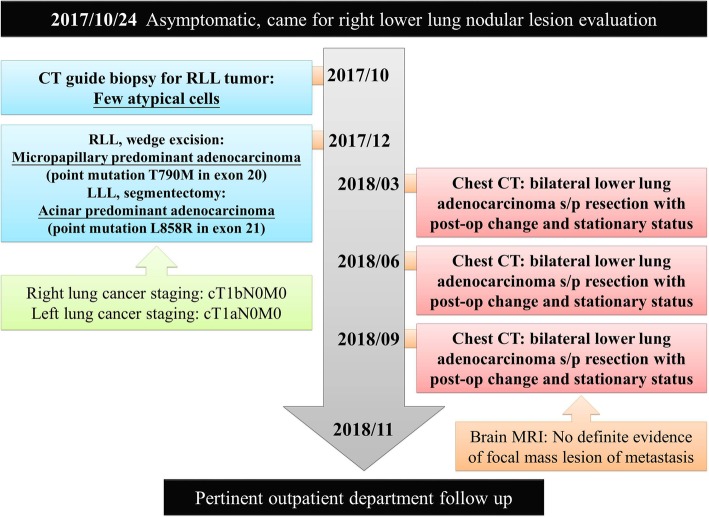
The follow-up timeline

A 68-year-old Taiwanese male never-smoker with no family history visited our hospital due to a tumor in the right lower lung identified in a chest radiograph. He mentioned that the tumor had been detected more than 5 years previously during a health examination; however, he ignored the problem because the clinician at that time stated that the lesion was highly likely to be benign. We performed chest computed topography (CT), which showed a well-defined tumor in his right lower lung with central calcification (Fig. 1 a, b), while another faint nodular lesion with an irregular border and ground glass pattern was detected over the left lower lung field (Fig. 1 c, d). A CT-guided biopsy was performed of the right lower lung tumor, and the pathologic examination showed the presence of atypical cells and positive staining of TTF-1 and CK7. A surgical intervention was then suggested to make a definite diagnosis. The results disclosed micropapillary predominant adenocarcinoma (Fig. 2a). Accordingly, the left lower lung nodule was resected to allow for a complete pathological staging. The left lower lung nodule showed acinar-predominant adenocarcinoma (Fig. 2b). Both were positive for TTF-1 but negative for ALK (D5F3) and p40. Real-time polymerase chain reaction (PCR) analysis showed that the right lower adenocarcinoma had an epidermal growth factor receptor (EGFR) mutation presenting as point mutation T790M in exon 20, while the left lower lung adenocarcinoma had a point mutation in exon 21 of EGFR (Fig. 3 a, b). A whole body bone scan and positron emission tomography (PET) scan were performed, which showed negative findings for metastatic lesions. The patient remains under regular follow-up with no complications or limitations in daily life. There was also no detectable tumor growth in the year after the diagnosis (Fig. 4).

## Discussion

In spite of the low incidence of SMPNSCLC in NSCLC [1, 8], earlier detection and surgical intervention have been shown to be beneficial for patient outcomes [9]. Lung cancer is associated with the highest mortality rate among various cancers worldwide, including Taiwan. In recent years, advances in CT technology requiring an increasingly low dose for lung examinations have led to the earlier discovery of lung cancer. Consequently, a higher rate of multiple lung nodules has been detected on chest CT scans. Once a solid lung mass has been identified, and other lobes or segments have more than one solid nodules, SMPNSCLC should be considered. Otherwise, if an initial CT-guided biopsy via the right lower lung mass shows atypical cells, which cannot fully exclude malignancy in spite of the high probability of the lesion being benign through concentric calcification via the CT scan [10, 11], intensive surgical intervention is suggested [1, 12]. In our case, we performed prompt surgical resection of the lung tumor. The different EGFR mutations in the two lung adenocarcinomas prompted us to investigate the tumor origin, and both tumors presented as treatment-naïve mutants occurring synchronously.

EGFR mutations have a good response to EGFR-tyrosine kinase inhibitors (TKIs), which are widely prescribed to treat lung cancer [13]. However, acquired resistance of the T790M point mutation in exon 20 after TKI treatment is common and has been reported in around 50% of tumors that fail treatment [14].

Treatment-naïve NSCLC with the EGFR T790M mutation is rare, and it has only been reported in around 2% of patients with EGFR-mutant lung cancer [15]. Germline T790M mutations were found up to 50% of treatment-naïve NSCLC. When germline EGFR T790M ‘drug–resistance’ mutation is associated with familial NSCLC, it seemed to occur in cis with the T790M mutation [16].

These patients were thought to refer for genetic counseling for germline screening of their relatives. Recent studies have reported increased numbers of a small proportion of “pretreatment T790M mutations” when using ultra-sensitive detection methods such as droplet digital polymerase chain reaction. In addition, an increase in the detection rate has been shown to be proportional to tumor size and tumors with common EGFR-activating mutations [17]. Accordingly, the prevalence of complex mutations including the classical (del19 + L858R) mutation or various “co-occurring” EGFR mutations may be higher than previously thought [18–22]. Concurrent mutations in exon 20 (T790M) and L858R have been reported in around 0.9% of EGFR mutation-positive patients before exposure to EGFR-TKIs, and in around 24% of compound EGFR-mutated lung cancers [21, 22]. The origin of treatment-naïve T790M mutations may be derived from somatic or germline EGFR gene mutations [23, 24].

EGFR mutations have also frequently been reported in patients with multiple primary lung cancer, and complicated mutations (L858R/S768I, L858R/20ins, G719X/T790M, 19del/T790M, 19del/L858R, and 19del/20ins) have only been found in female patients [25]. Our male patient was an unusual case of early-stage synchronous double primary treatment-naïve EGFR-mutant NSCLC with de novo T790M and L858R mutations in each tumor. Our case was confirmed as stage I SMPNSCLC (left lung: T1aN0M0, right lung: T1bN0M0), and thus the third-generation irreversible TKI osimertinib was not indicated [26]. Nevertheless, to date, osimertinib is considered to take priority over standard EGFR-TKIs in the first-line treatment of EGFR mutation-positive advanced NSCLC [27]. There are two main findings. First, the right lung mass was misdiagnosed as a benign lesion before ultimately proving to be lung adenocarcinoma, highlighting the importance of aggressive surgical intervention to obtain tissue proof in the early phase. Second, to the best of our knowledge, treatment-naïve T790M and L858R mutations in separate tumors diagnosed at the same time has not previously been reported. This illustrates that various kinds of EGFR mutations can occur in different lobes simultaneously and that they may be present from the very beginning [25]. The presence of complex mutations shows that tumor heterogeneity can exist in one tumor sample and also different tumors at the same time.

## Conclusion

This case highlights that early screening for lung cancer either in smokers or never smokers is important. Tumors resembling a benign pattern with central calcification depending on the morphological method may be misdiagnosed as lung malignancy. Although an accurate preoperative diagnosis can be challenging, in our opinion, intensive efforts to make a diagnosis through surgical resection or biopsies to allow for tailored optimal treatment may be preferential for the best patient outcomes.

## Data Availability

As a case report, all data generated or analyzed are included in this article.

## Abbreviations

AIS: Adenoma in situ
CT: Computed topography
EGFR: Epidermal growth factor receptor
MIA: Minimally invasive adenocarcinoma
MMPL: Metachronous multiple primary lung cancer
MPLC: Multiple primary lung cancer
SMPNSCLC: Synchronous multiple primary non-small cell lung cancer
TKI: Tyrosine kinase inhibitors
